# Influence of injection rates of calibrating standard solution on monitoring pulse indicator continuous cardiac output

**DOI:** 10.1186/s12938-018-0453-0

**Published:** 2018-03-16

**Authors:** Chen Shu-Lan, Lan Fang-Chen, Du Zhen-Shuang, Xu Ya-Ping, Zhao Hui-Ming, Zeng Cui-Ping, Miao Yu

**Affiliations:** General Surgery Department, The 180th Hospital of Chinese People’s Liberation Army, Quanzhou, 362000 Fujian China

**Keywords:** Calibrating standard solution, Pulse indicator continuous cardiac output, Injection rate

## Abstract

**Objective:**

This study aimed to investigate the influence of injection rates of calibrating standard solution on monitoring pulse indicator continuous cardiac output (PICCO, made in Germany), and thereby to provide significant references for clinical practice.

**Methods:**

A total of 108 critical patients in stroke intensive care unit were identified. All these participants received transesophageal cardiac color Doppler ultrasound, and within 15 min PICCO equipment was utilized to monitor the relevant parameters, by means of 0 °C calibrating standard solution, and the injection speeds were 2–4, 5–7, and 8–10 s. Besides, the monitoring indicators were as follows, cardiac index, global ejection fraction, global end diastolic volume index. The potential correlations were evaluated between PICCO and transesophageal cardiac color Doppler ultrasound.

**Results:**

All the data was available, and the monitored parameters of PICOO at 2–4, 5–7, and 8–10 s were positively correlated with the parameters obtained from transesophageal cardiac color Doppler ultrasound (*P* < 0.05). Specially, it is worth emphasizing that the best correlation between them could be provided when the injection rate was 2–4 s.

**Conclusion:**

When the injection rate at 2–4 s, the parameters obtained by PICOO were much closer to that of transesophageal cardiac color Doppler ultrasound. Furthermore, the parameters of PICOO obtained at 2–4 s could better reflect cardiac function of patients.

## Background

Being regarded as a novel combination technology in medical domain, and one less invasive thermodilution-based technique, pulse indicator continuous cardiac output (PICCO) equipment could not only supervise pulse indicator continuous cardiac output, but also monitor lung temperature dilution cardiac output [1–4]. Recently, this technology could be utilized to monitor the conventional hemodynamic parameters, in order to provide significant references for critically ill patients [2–5]. PICCO surveillance (PICCO Plus; PULSION Medical Systems, Munich, Germany) has plenty of advantages, including minimal trauma, lower risk, convenience, precision, etc. [6, 7]. Therefore, this technique could be applied to comprehensively monitor cardiovascular functional status, the preload and after load of heart, cardiac systolic function, lung water [3, 8, 9], which is good evidence regarding the efficacy of goal-directed fluid management based on transpulmonary thermodilution-measured variables using the PICCO monitoring system [10]. In further, PICCO can estimate extravascular lung water and the pulmonary vascular permeability index, which may be used to assess the severity of pulmonary dysfunction [11]. The PICCO monitor is an all-inclusive device, which provides a full picture of hemodynamic status.

And the detailed monitoring methods are as follows, firstly, ice salt water at 0 °C is injected into superior vena cava, and then the continuous parameters can be calculated via analyzing area under the curve of arterial pressure waveform [12, 13]. In our hospital, this kind of monitoring technique is commonly utilized for patients with cardiac diseases, and we attempted to assess the specific situation of PICCO. Nevertheless, the injection rate of calibrating standard solution can be consistent with each other, and thereby results in the inevitable error of monitored parameters [14]. Hence, this research aimed to investigate the better injection rate of calibrating standard solution, in order to more precisely monitor the PICCO parameters.

## Materials and methods

### General information

This research was approved beforehand by the institution ethics committee in our hospital. According to the relevant regulations of ethics, the informed consent of patients had been obtained before investigation [15, 16]. 108 critically ill patients in the stroke intensive care unit (SICU) were included in this research. In detail, 24 patients suffered from traumatic shock, 49 patients with septic shock, 19 patients were identified on account of hypovolemic shock, 9 cases suffered from multiple organ dysfunction syndrome (MODS), 7 cases with acute renal failure. And, there were 68 males and 40 females, their ages ranged from 19 to 74, and the average age was 40 ± 11.4.

## Methods

These included participants were assigned on the basis of matched pair design. With 24 h, they all received transesophageal cardiac color Doppler ultrasound according to the standardized operation procedures. Specifically, by means of a triplicate injection of 15 ml ice-cold 0.9% saline administered through a temperature detecting inline sensor central vein catheter, cardiac output was evaluated by thermodilution. A femoral or brachial artery catheter registers the time until the bolus attains and identifies the alteration of temperature. The PICCO PLUS system applied in this research belongs to a class of biomedical device [17, 18].

And the detailed operation methods were described as follows. The patients in the general anesthesia underwent tracheal intubation to keep breath steady, and the dual-rate probe with 5 MHz frequency was inserted into esophageal cavity through oral cavity. Once the distance between incisor and probe reached 35 cm, the probe turned to the rear of heart, and the color echocardiography was connected outside with the probe. Afterwards, the 0 °C ice saline was used as the calibrating standard solution (15 ml in total), with the purpose of monitoring the various parameters of PICCO. In detail, the injection rates were 2–4, 5–7, and 8–10 s, respectively.

### Monitoring indicators

#### PICCO monitor

After admission into SICU, those patients who met the inclusion criteria were informed the treatment and their own condition. After informed consent of patients or their immediate family members, the patients received the right central vein catheterization, and then the t-branch pipe combined the syringe, cardiac output (C) module, and temperature sensor of interface cable. Besides, the specialized artery monitor catheter (Model number: PV2014L16N; Manufacturer: PUSTON in Germany) which was implanted through femoral artery was connected with CO module, connector interface, pressure sensor, and pressure module (Model number: PV8115; Manufacturer: PUSTON in Germany). When the measuring program started, ice saline was injected into central vein at the speed of 15 ml per time [19]. The computer obtained continuous parameters by analyzing the area under the thermal dilution method and the arterial pressure waveform curve.

#### GEF, GEDI, and CI monitor

To monitor global ejection fraction (GEF), global end diastolic volume index (GEDI), and cardiac index (CI), the central venous infusion was temporarily paused for at least 30 s, and then the PICCO equipment was calibrated. Firstly, the CVP value was imported into PICCO monitoring instrument. Secondly, the 0 °C ice saline was injected at the speed of 2–4 s, and the average value was calculated on the basis of three tests results. After 5 min, ice saline was resupplied at the speed of 5–7 s, and the average data was obtained according to three repeated tests. After 5 min, ice saline was injected again at the speed of 8–10 s, and the average value was calculated as mentioned above.

## Statistical analysis

SPSS19.0 software was utilized to perform data analysis, and all the data was illustrated as mean ± standard deviation. The paired bilateral t test was then carried out hypothesis testing, and then the linear regression adopted to make correlation analysis. There was statistically significant when P value was < 0.05.

## Results

### Comparison of GEF, GEDI, and CI between groups

**Fig. 1:**
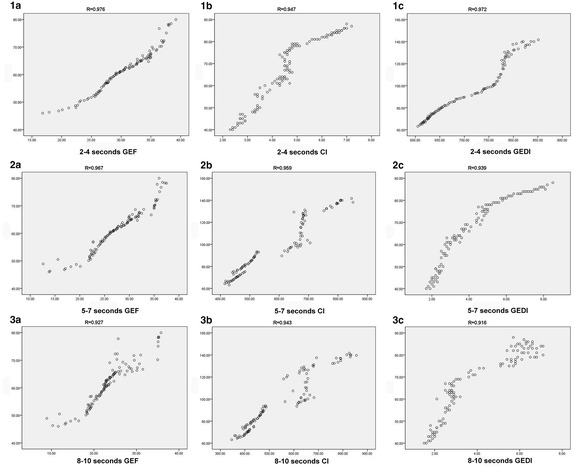
Correlation analysis between transesophageal heart color ultrasound monitor and PICCO parameters. **1a** Monitoring data of global ejection fraction (GEF) in 2–4 s; **1b** Monitoring data of cardiac index (CI) in 2–4 s; **1c** Monitoring data of global end diastolic volume index (GEDI) in 2–4 s; **2a** Monitoring data of global ejection fraction (GEF) in 5–7 s; **2b** Monitoring data of cardiac index (CI) in 5–7 s; **2c** Monitoring data of global end diastolic volume index (GEDI) in 5–7 s; **3a** Monitoring data of global ejection fraction (GEF) in 8–10 s; **3b** Monitoring data of cardiac index (CI) in 8–10 s; **3c** Monitoring data of global end diastolic volume index (GEDI) in 8–10 s

After comprehensive data analysis, the statistical results are demonstrated in Tables 1 and 2. As shown in Fig. 1, the comparisons of PICCO monitor parameters at the drip speed of 2–4, 5–7, and 8–10 s were illustrated, respectively. And the difference was statistically significant (*p* < 0.05). After comparison, it can be speculated that the PICCO parameters at the drip speed of 2–4 s could offer better indicator of patients situation.

**Table 1:** Comparison of PICCO monitor parameters at the drip speed of 2–4, 5–7, and 8–10 s

	2–4 s	5–7 s	8–10 s	Normal value	F	*P*
GEF	30.35 ± 4.87	27.47 ± 5.47	23.28 ± 5.32	25–35	49.113	< 0.001
GEDI	713.36 ± 73.67	585.75 ± 126.94	545.53 ± 140.37	680–800	58.660	< 0.001
CI	4.61 ± 1.28	4.32 ± 1.87	4.02 ± 1.81	3.0–5.0	3.088	= 0.047

**Table 2:** Whole body ejection fraction (GEF), systemic end-diastolic volume (GEDI), cardiac index (CI), which were all consistent with color Doppler surveillance

			Color ultrasonic diagnosis		KAPPA	*P*
			Normal	Abnormal		
Injection rate 2–4 s	GEF	Normal	78	0	0.771	< 0.001
		Abnormal	9	21		
	GEDI	Normal	50	2	0.907	< 0.001
		Abnormal	3	53		
	CI	Normal	64	1	0.761	< 0.001
		Abnormal	9	34		
Injection rate 5–7 s	GEF	Normal	60	0	0.464	0.048
		Abnormal	27	21		
	GEDI	Normal	17	6	0.214	0.007
		Abnormal	36	49		
	CI	Normal	39	0	0.426	< 0.001
		Abnormal	34	35		
Injection rate 8–10 s	GEF	Normal	19	7	− 0.055	0.269
		Abnormal	68	14		
	GEDI	Normal	7	4	0.06	0.308
		Abnormal	46	51		
	CI	Normal	12	0	0.113	0.011
		Abnormal	61	35

### Whole body ejection fraction (GEF), systemic end-diastolic volume (GEDI), cardiac index (CI)

As shown in Table 2, the data obtained from 2 to 4 s was highly consistent with the results provided by ultrasonography. But, the data obtained from 5–7 to 8–10 s was not highly consistent with that acquired from ultrasonography, the consistency between them was not good.

### Correlation analysis between transesophageal heart color ultrasound monitor and PICCO parameters

As shown in Fig. 1, the PICCO parameters gained at the drip speed of 2–4 s were positively correlated with the results provided by transesophageal cardiac color Doppler ultrasound, and the correlation coefficient appeared much higher when compared with the data obtained at the injection speed of 5–7 s and 8–10 s.

## Discussion

The Blood flow surveillance includes invasive monitoring, minimally invasive monitoring, noninvasive monitoring. In detail, the invasive hemodynamic monitoring refers to measure central venous pressure (CVP) by means of Swan-Ganz catheter, in order to assess cardiac function indicators. Nevertheless, these indicators are not only associated with cardiac volume, but also can be influenced by myocardial compliance, chest pressure and other potential factors [20–23]. Therefore, the invasive hemodynamic monitoring can not reflect the cardiac volume in the filling period. Besides, the external Swan-Ganz catheter can probably lead to various serious complications, such as cardiac mechanical damage, ventricular rhythm Abnormal, pulmonary embolism, pulmonary artery rupture, etc. [24]. Meanwhile, the non-invasive monitoring can not result in serious tissue damages, and the operation method appears more convenient. But, the detection accuracy remains relatively poor. On the contrary, the parameters which are provided by PICCO monitor, such as cardiac index (CI), global ejection fraction (GEF), global end diastolic volume index (GEDI), can not be influenced by these factors mentioned above. Therefore, PICCO monitor is able to monitor hemodynamic parameters in real time manners, and can better reflect the changes of cardiac functions [25–27].

As far as we are concerned, PICCO is a minimally invasive hemodynamic monitoring technology, with the advantages of repeatable, sensitive, simple, etc. Hence, this Monitoring technique can provide comprehensive hemodynamic monitoring parameters, and can reflect cardiac contractile function more accurately. When compared with conventional monitor method, PICCO also possess much more advantages, including simple operation, longer time of indwelling catheter, convenient observation and nursing. Before the formal monitor, the PICCO calibrating standard solution should be diluted for at least three times, and the final temperature of calibrating standard solution is 0 °C [28]. Currently, it has not been proved about the drip speed of calibrating standard solution. For instance, Jiang et al. [29] suggested that the solution should be injected within 4 s, Cao [30] suggested that the standard solution should be injected within 7 s, and Li [31] suggested it within 10 s. After comprehensive data analysis, it can be illustrated that the majority of searchers did consider that the calibrating standard solution should be injected within 4 s, and a fraction of them did not mention the specific injection speed. Meanwhile, it has been acknowledged that the different drip speed of calibrating standard solution can cause the unavoidable errors. And, on the basis of results in this research, we conclude that PICCO monitor can provide the corresponding parameters when the calibrating standard solution is titrated at the speed of 4–10 s. In this study, we attempt to compare three different injection rates of calibrating standard solution. Firstly, a total of 108 critically ill patients in the stroke intensive care unit (SICU) were included in this research. Secondly, all these identified patients received PICCO monitor. In detail, the ice salt solution at 0 °C was injected into superior vena cava, at the drip speed of 2–4, 5–7 and 8–10 s, respectively. After data analysis and comprehensive comparison among them, as shown in Fig. 1 and Table 1, these various parameters obtained from PICCO monitor were assessed on the basis of paired-samples t-test, and the statistical differences among 2–4, 5–7 and 8–10 s were significant (*P* < 0.05). Besides, as shown in Table 2, the data obtained from 2 to 4 s was highly consistent with the results provided by ultrasonography. But, the data obtained from 5–7 to 8–10 s was not highly consistent with that acquired from ultrasonography, the consistency between them was not good. In addition, the PICCO parameters gained at the drip speed of 2–4 s were positively correlated with the results provided by transesophageal cardiac color Doppler ultrasound, and the correlation coefficient appeared much higher when compared with the data obtained at the injection speed of 5–7 and 8–10 s.

The calculation method of PICCO PLUS system (Manufacturer: PULSION company) refers to the combination of average transmission time of thermal dilution curve and exponential descent time. When the measurement based on the principle of thermodilution was carried out, the quantitative cooling solution should be injected into vein as soon as possible, and its temperature should below the blood temperature at least 10 °C. In addition, the occurrence time of PICCO monitoring curve shifted to an earlier time point, when the ice salt solution at 0 °C was injected at the drip speed of 2–4 s, and the crest appeared higher. On the contrary, when the ice salt solution was injected at the speed of 5–7 s and 8–10 s, the occurrence time of PICCO monitoring curve shifted to a later time, and the crest appeared lower. Therefore, the PICCO parameters obtained at the injection rate of 2–4 s were more close to the indicators provided by transesophageal cardiac color Doppler ultrasound (as shown in Table 2). Namely, when the 0 °C calibrating standard solution was injected at the speed of 2–4 s, the PICCO parameters appeared more valuable.

## Conclusion

In summary, according to the KAPPA test and paired-samples t-test between PICCO parameters and transesophageal cardiac color Doppler ultrasound, it can be speculated that the PICCO parameters obtained from 0 °C calibrating standard solution at the injection rate of 2–4 s were much more valuable to reflect various clinical indicators. In consideration of the higher safety and credibility of PICCO monitoring method, as well as the steadily rising popularity in clinical practice, how to correctly obtain and accurately measure the parameters is the most important and essential factor. Therefore, it can be suggested that PICCO monitoring could offer a more rational guide for critical patients in stroke intensive care unit, and the calibration solution at the injection rate of 2–4 s could better reflect cardiac function indicators of patients. Nevertheless, its role in this aspect warrants further investigation.
